# Development and psychometric evaluation of "Caring Ability of Mother with Preterm Infant Scale" (CAMPIS): a sequential exploratory mixed-method study

**DOI:** 10.1186/s12912-024-01960-7

**Published:** 2024-04-29

**Authors:** Saleheh Tajalli, Abbas Ebadi, Soroor Parvizy, Carole Kenner

**Affiliations:** 1 School of Nursing and Midwifery, Tehran University of Medical Sciences, Tehran, Iran; 2 Behavioral Sciences Research Center, Life Style Institute, Nursing Faculty, Baqiyatallah University of Medical Sciences, Tehran, Iran; 3 Nursing and Midwifery Care Research Center, Pediatric Nursing Department, School of Nursing and Midwifery, Iran University of Medical Sciences, Tehran, Iran; 4 Center for Educational Research in Medical Sciences (CERMS), Department of Medical Education, School of Medicine, Iran University of Medical Sciences, Tehran, Iran; 5 School of Nursing and Health Sciences, The College of New Jersey, Ewing, NJ USA

**Keywords:** Caring ability, Maternal caregiving, Caregivers, Preterm infant, Psychometric, Scale, Neonatal

## Abstract

**Background:**

Caring ability is one of the most important indicators regarding care outcomes. A valid and reliable scale for the evaluation of caring ability in mothers with preterm infants is lacking.

**Objective:**

The present study was conducted with the aim of designing and psychometric evaluation of the tool for assessing caring ability in mothers with preterm infants.

**Method:**

A mixed-method exploratory design was conducted from 2021 to 2023. First the concept of caring ability of mothers with preterm infants was clarified using literature review and comparative content analysis, and a pool of items was created. Then, in the quantitative study, the psychometric properties of the scale were evaluated using validity and reliability tests. A maximum likelihood extraction with promax rotation was performed on 401 mothers with the mean age of 31.67 ± 6.14 years to assess the construct validity.

**Result:**

Initial caring ability of mother with preterm infant scale (CAMPIS) was developed with 64 items by findings of the literature review, comparative content analysis, and other related questionnaire items, on a 5-point Likert scale to be psychometrically evaluated. Face, content, and construct validity, as well as reliability, were measured to evaluate the psychometric properties of CAMPIS. So, the initial survey yielded 201 valid responses. The three components: 'cognitive ability'; knowledge and skills abilities'; and 'psychological ability'; explained 47.44% of the total observed variance for CAMPIS with 21 items. A subsequent survey garnered 200 valid responses. The confirmatory factor analysis results indicated: χ2/df = 1.972, comparative fit index (CFI) = 0.933, and incremental fit index (IFI) = 0.933. These results demonstrate good structural, convergent, discriminant validity and reliability. OMEGA, average inter-item correlation (AIC), intraclass correlation coefficients (ICC) for the entire scale were at 0.900, 0.27 and 0.91 respectively.

**Conclusion:**

Based on the results of the psychometric evaluation of CAMPIS, it was found that the concept of caring ability in the Iranian mothers with preterm infants is a multi-dimensional concept, which mainly focuses on cognitive ability, technical ability, and psychological ability. The designed scale has acceptable validity and reliability characteristics that can be used in future studies to assess this concept in the mothers of preterm infants.

**Supplementary Information:**

The online version contains supplementary material available at 10.1186/s12912-024-01960-7.

## Introduction

In recent years, the technology advancements in the field of caring for mothers during pregnancy and delivery, and their infants resulted in increased survival of preterm infants [1]. According to the assessment of the world health organization (WHO), the prevalence of preterm birth globally is 10.6% [2].

Preterm birth is often an unexpected event for women [3], and hospitalization in the NICU is considered unavoidable [4]. The birth of a preterm infant and hospital stay in the NICU is very stressful for parents. The source of this stress includes the medical condition and parental separation from their infant. Mother-infant separation often results in the feelings of anxiety, fear, depression thus potentially decreasing the mother’s sensitivity to infant cues, which in turn, may delay infant development [5]. Many mothers experience acute stress disorder, which can last from 3 days to 1 month. It can be associated with symptoms such as troublesome memories, frequent and annoying restlessness and sadness, as well as post-traumatic stress [6].

The feeling of loss is a source of suffering and grief for the mothers of preterm infants. In the initial days following birth, the primary focus of parents is often on the infant's chances for survival. However, as time passes, this preoccupation can evolve into a potential cause of grief, anxiety, and feelings of culpability (related to an incomplete pregnancy)[6]. Therefore, in some cases, the mothers cannot focus on their preterm infants. Becoming a mother requires reorganizing and changing one’s personal identity. Mothers must change their identity from being a daughter and a wife in the generation to which they belong to being a mother for the next generation. They start this process from the pregnancy period, initially fantasizing about motherhood [7]. However, at the end of the pregnancy period, with the birth of a preterm infant, this process stops. In fact, preterm birth is a sudden stop in the process of mother's self-representation [8]. Consequently, the experience of transitioning into motherhood for women who give birth prematurely may differ from that of others. This is due to the fact that the psychological readiness and preparation required for assuming the role of a mother comes to an abrupt halt upon the premature birth of their infant. As a result, these women are forced to quickly adapt to their new circumstances [9].

Review of literature shows caring ability concept have three dimensions: cognitive, knowledge and patience [10]. Published caring ability scales evaluate ability of take care in informal caregiver of patient with cancer named ‘caring ability of family caregivers of patients with cancer scale (CAFCPCS)’ [11] or professional care giver named ‘The caring ability inventory’[10]. Also, published tools, focused on others aspects of take caring ability. So family empowerment tool named ‘Family Empowerment Scale’ [12], asses the empowerment of family members of patients with chronic disease as an outcome of taking care empowerment. The parent Engagement tool is another scale named ‘parent risk evaluation and engagement model and instrument (PREEMI)’ that evaluates parent risk evaluation and engagement of mothers with preterm infants [13]. The discharge preparedness scales were ‘perceived readiness for discharge after birth scale (PRDBS) and ‘Parent discharge readiness’ [14], that evaluate readiness for discharge. These evaluate postpartum mother’s perceptions of readiness for discharge from the hospital that was adapted from a scale measuring adult and elderly postsurgical patients’ perceptions of their readiness for discharge. Also assert for readiness is different from the ability to continue taking care after discharge. The scales 'Perceived maternal parenting self‐efficacy (PMP S‐E)' and 'Self‐efficacy in infant care scale' [15, 16] were developed to assess the caregiver's belief in their ability to effectively care for their child, with a specific emphasis on self-confidence. These scales aim to provide a means of measuring the caregiver's expectations regarding the outcomes of their caregiving efforts. Although our previous study show a mother with optimal caring ability has sufficient cognitive ability, technical ability, and psychological ability [17]. This is a clear gap in the designed scale that could be overcome by a new scale that focuses take caring ability of mother with preterm infants. To sum up, there is no scale developed for the caring ability of mothers with preterm infants; therefore, the purpose of this study is to examine the real-life experiences of mothers with preterm infants and other professional and family. Caregivers, who have directly experienced taking care of preterm infants in order to then, design and perform the psychometric evaluation of a tool to assess the caring ability of mothers with preterm infants.

The purpose of this study was to develop a preterm caring ability scale and to examine its psychodynamic properties in mother of preterm infants with gestational age less than 32 weeks.

## Method

This study utilized a mixed-method exploratory design to develop and psychometric evaluate the caring ability of mother with preterm infant scale (CAMPIS) from July 2021 to October 2023. The research involved both mothers and professionals caring for preterm infants. The research consisted of two main phases: first, a qualitative study was conducted to generate the scale items, followed by a quantitative approach to evaluate the psychometric properties of the scale.

### First step: qualitative study and item generation

The purpose of this step was to explain the concept of caring ability in the mothers of preterm infants, and to create a set of items to design the target scale. This step involved the identification of concepts through literature review published from 1995 to 2020 [17]*.*

In the second step, 18 semi-structured individual interviews of 60–80 min were organized to understand better the caring ability of mother with preterm infant concept. The face-to-face semi-structured interviews were conducted in a serene setting, either in the hospital room or the participants' home, as per their request and preference. The interviews involved mothers, grandmothers, and fathers, and were carried out without the presence of any other individuals. Due to the coronavirus social distance limitation, the interviews with the physicians and the nurses were conducted online and also recorded via Skyroom software.

We decided to finish the interview, when we interviewed the 18 participants, the information he/she provided is similar to those provided by the former ten participants (data saturation). Totally, 11 mothers, 2 fathers, 2 grandmothers of preterm infants, 1 neonatal nurse, and 2 neonatologists working in these wards participated in the present study. The participants were residents in the NICU in 5 hospitals in Tehran, Iran, representing a range of ages, genders, and caring role. The interview process started with a general question such as "Would you please explain mothers’ ability to take care of a preterm infant?", "In which situations do you feel she are more capable?", and "Which factors decreased her ability?" Then, based on the participant’s responses, the interviews continued with exploratory questions such as "Could you please explain more? or “Could you give an example in this regard?".

All participants were interviewed individually and each interview lasted between 60 and 80 min. The texts of the interviews were analyzed using the using Lindgern et al. [18] approach by MAXQDA software version 10. Qualitative interview resulted in initial items generation.

### Second step: quantitative study and CAMPIS psychometric properties evaluation

#### Face validity

Face validity was evaluated through qualitative and quantitative approaches. In the qualitative approach, the scale was sent to 10 mothers of preterm infants, they were asked to evaluate the scale in terms of difficulty, relevance, and ambiguity. The participants assessed the items based on their own judgment, ensuring that they were able to understand them. In order to further evaluate the suitability of the items, five additional mothers were included in the quantitative approach. These mothers were asked to rate the items on a 5-point Likert scale, ranging from completely suitable to not suitable at all. The impact score was calculated through the following equation: impact score = frequency (%) × appropriateness. A score above 1.5 was considered acceptable [19].

#### Content validity

The content validity of CAMPIS was evaluated through quantitative and qualitative approaches. In the qualitative approach, the scale was distributed among 22 neonatal nursing specialists, neonatal subspecialists, and scale development specialists to evaluate the items in terms of grammar and wording, item allocation, and scaling.

Then the content validity of the scale was modified by measuring content validity ratio (CVR) and content validity index (CVI) to ensure that the scale measures the intended construct in two separate stages. So, the 26 specialists, as highly knowledgeable about the mother of preterm infant or scale development, were asked to evaluate the items regarding necessity and relevancy. In CVR, 12 specialists evaluated the necessity of CAMPIS on a 3-point Likert scale (1 = not necessary, 2 = useful but not necessary, and 3 = necessary). CVR was calculated through the following formula: [ne – (N/2)]/(N/2), where “ne” is the number of the experts who rate the items as “essential”, and N is the total number of the items. The result was interpreted using Lawshe's content validity ratio [20].

Following the implementation of the required modifications based on the feedback provided by experts, the effectiveness of CAMPIS was further evaluated by 14 additional specialists in terms of the CVI. This evaluation was conducted using a four-point Likert scale, where a score of 1 indicated irrelevance, 2 denoted relative relevance, 3 represented relevance, and 4 signified complete relevance. The I-CVI, Kappa statistic, and S-CVI/Ave were computed to assess the content validity index at both the item-level and scale-level. A Kappa value exceeding 0.75 was deemed indicative of excellent agreement [19].

#### Item analysis

Before construct validity of the structure, the items were analyzed to identify the possible problems. At this step, 48 mothers of preterm infants, with the mean age of 31.88 ± 5.9 years, were selected through convenience sampling and enrolled. They were asked to identify that there were problems such as inappropriate reverse questions. Also they were asked to completed the hardcopy of CAMPIS and item-total correlations was evaluated for some items. A correlation coefficient lower than 0.32 or above 0.9 was considered as the criteria for removing the items [19].

#### Participants

The sample included the mothers of the preterm infants with a gestational age < 34 weeks. The criteria for entering the study consisted of the infant’s having been hospitalized in the NICU for more than two weeks, the infant’s not suffering from any major congenital anomalies, giving consent to participate in the study, and being able to use social networks such as WhatsApp. Based on the rule of thumb, that is, which considers 200 participants as an appropriate sample size [19], 401 mothers were considered for two phases at this step: 201 for the exploratory factor analysis (EFA) assessment, and 200 for the confirmatory factor analysis (CFA).

The participants were selected using convenience sampling through being in the hospital on the day of discharge, membership in the social groups related to following up mothers with preterm infants discharged from the NICU, and recommendations. In this step, data was collected online. For this purpose, an online questionnaire was created through the Porsline form, and its URL link (https://survey.porsline.ir/s/d8uW4Jp) was sent to the participants through the Telegram or WhatsApp social network applications (as the most common social networks among Iranian users).

#### Measures

The questionnaire used in this step included two parts. The first part was related to the infant's demographic characteristics, such as the infant's gender, and gestational age, and the mother's demographic characteristics, such as the mother's age, the infant's age, the mother's education, mothers' parity, assisted reproductive methods, the type of delivery, and previous experience in caring for infants. The second part included initial CAMPIS, with 38 items, for measuring the concept of the caring ability of mothers with preterm infants, on a five-point Likert scale (1 = never to 5 = always).

#### Construct validity

The construct validity of this scale was evaluated using EFA and CFA by SPSS 26 (SPSS Inc., Chicago, IL, USA). According to normal distribution of variables (skewness of ± 3, kurtosis of ± 7 and Mardia's coefficient less than 20) EFA was evaluated through Maximum likelihood factor analysis using Promax rotation. In addition, Kaiser–Meyer–Olkin (KMO) and Bartlett tests were used to estimate the adequacy and the appropriateness of the sample. KMO values higher than 0.9 were interpreted as excellent [19]. In order to extract the factors according to Thompson and Daniel recommendation [21] multivariate approach was used to identify the number of factors to extract in the EFA.

Then the factors were analyzed using the method of maximum likelihood analysis, which is one of the most common methods of data reduction. At first, 5 factors had an eigenvalue greater than 1, but considering that 3 factors explained an eigenvalue greater than 1.5 and a variance greater than 5%.

To extract the factor structure, exploratory graph analysis was used. The actual values ​​of the matrix were compared with the randomly generated matrix. The number of the components which have a higher variance in comparison with the components obtained from random data, after successive repetitions, is considered as the correct number of factors for extraction [22]. A factor loading of approximately 0.3 was considered to determine the presence of an item in a latent factor, and the items with communalities < 0.2 were excluded from EFA. The factor loading was estimated using the following formula: CV = 5.152 ÷ √ (n—2), where CV is the number of the extractable factors, and N is the sample size.

In the next step, the factor structure determined by EFA was evaluated by CFA. For this purpose, CFA was evaluated using Maximum likelihood factor analysis and the most common goodness-of-fit indices using SPSS/AMOS 26 software [22].

To discuss the fitness of the model on CFA, we can consider the various criteria for model fit indices. It has been suggested that Root Mean Square Error of Approximation (RMSEA) values less than 0.05 are good, and values between 0.05 and 0.08 are acceptable [23]. Therefore, the RMSEA value of 0.059 in this sample indicates an acceptable fit. The goodness of fit index (GFI) value of this sample, 0.88, is below 0.9, but the GFI is known depending on the sample size [24]. The frequency interference index (RFI) value, 0.085, is close to 0.9, which shows a relatively good fit [25]. The other fit indices, comparative fit index (CFI), incremental fit index (IFI), and Tucker-Lewis index (TLI), should be over 0.9 and parsimonious normed fit index (PNFI) should be over 0.5 for a good fit [25].

#### Reliability

The reliability was evaluated using internal consistency, stability, and absolute reliability approaches with SPSS 26. The internal consistency was evaluated using, McDonald's omega (Ω), and average inter-item correlation (AIC). The McDonald Omega of 0.7 or above, and the AIC of 0.2 to 0.4 were considered as acceptable criteria to evaluate the internal consistency. The consistency of CAMPIS was evaluated through calculating intraclass correlation coefficients (ICC) using a two-way random effects model [19]. The retest method with a time interval of 72 h (the day before discharge and 72 h after discharge) was used in 48 mothers with preterm infants. An ICC value > 0.8 is considered as the acceptable value for stability. In addition, the absolute reliability was evaluated using the standard error of measurement with the formula Standard Error of Measurement (SEM) = SD √ (1- ICC).

Finally, the responsiveness was evaluated using minimum detectable change (MDC) with the formula MDC = SEM × Z × √ 2, and minimal important change (MIC) with the following formula: MIC = SD × 0.5. If the MIC is smaller than the MDC, the scale is responsive. Besides, the interpretability was evaluated through calculating the MDC and testing the hypothesis [22].

#### Ethical consideration

The present study was extracted from the nursing Ph.D. thesis fund by Nursing Care Research Center (NCRC), School of Nursing and Midwifery, Iran University of Medical Sciences, Tehran, Iran. All ethical considerations of the study were approved by the ethics committee at the University of Medical Sciences (IR. IUMS. REC.1398.1407).

## Result

In qualitative step a total of 43 articles were selected in the study after reviewing 23291 extracted articles. We explored attribute of take caring ability concept. Findings showed a mother with optimal caring ability has sufficient knowledge, high skills, a sense of sufficient self-efficacy [17]. This step resulted in 69 initial items generation.

Using the findings of the literature review, comparative content analysis, and other related questionnaire items, the research teammates designed the initial items to measure the caring ability of mothers with preterm infants. An example of item generation has been presented in Table 1. Then all the initial items (n: 104) were reviewed, and the generation of items was completed. In the selection of items, the focus was mainly on the features of the concept. The items of the pool were examined during joint meetings with the research team, and the items that were not in line with the purpose of this study were omitted according to experts’ opinions. First, repetitive descriptive items were deleted. For example, “I have access to healthcare providers and whenever I have questions, I can ask them” and “A healthcare provider answers my questions regarding the care of my child all day long 7 days a week.” This step resulted in 71 items. In the next step, the items with similar descriptions items were combined. For example, “I simply understand my child's needs.” and “When my child severely cries, I can figure out what my child wants.”

**Table 1:** An example of items generation from the codes

*Quotations*	**Condensed Meaning Units**	**Codes**	**Items**
*“… I saw color change due to the lack of oxygen while hospitalization…So, I can recognize if my infant is not breathing or is breathing with difficulty …”*	ability to recognize breathing stopes and distress	recognision highrisk apneas and distress breathing	I can recognize the signs and symptoms of shortness of breath and cyanosis
*“…My baby was born preterm although she is 1360 g, and the doctors said it is not enough for discharge, but I'm not afraid that my baby might be harmed after discharge, and I am ready enough for discharge…”*	to have enough feeling readiness for discharge to home	feeling readiness for discharge to home	I feel comfortable taking care of my baby at home I can balance life plans and care for my baby

Therefore, at this step, CAMPIS was developed with 64 items, descriptions about behaviors and attitudes, on a 5-point Likert scale (always, most of the time, sometimes, rarely, never) to be psychometrically evaluated. Face, content, and construct validity, as well as reliability, were measured to evaluate the psychometric properties of CAMPIS.

In quantitative study and CAMPIS psychometric properties evaluation phase the impact score evaluation of face validity step, score of all items were above 1.5, and was considered appropriate. According to degree of mothers’ judgement all items of CAMPIS were appropriate to assessment of take caring ability. Therefore, no item was deleted.

During the process of content validity, some items were modified according to panelist feedbacks. While evaluating content validity, in the qualitative approach, 8 items were merged into one item based on the suggestion of the expert panel. Regarding number of participants for CVR were 12, the minimum acceptable CVR score was 0.56. In this step, the CVRs of 7 items were less than 0.56 and removed. In the CVI assessment, the I-CVI for all the items was in the range of 0.83–1, the modified Kappa was in the range of 0.84–1, and the total S-CVI/Ave and S-CVI/UA were 0.93 and 0.40 respectively. In content validity step according to the results, the Kappa values of 8 items were lower than 0.75, so they were removed. Therefore, 23 items were eliminated and the total number of CAMPIS was reduced from 64 to 41 items in content validity evaluation step.

In the item analysis step, the item-total correlation for 3 items was 0.32 or less, therefore were removed. The final CAMPIS with 38 items entered the factor analysis step.

In construct validity step, totally 401 mothers with a gestational age of 34 weeks or less participated in the present study. Their infants did not have any major surgical problems/anomalies, and had been hospitalized in the NICU for more than two weeks. The mean age of the mothers was 31.77 ± 6.02 years, with the minimum age of 16 and the maximum of 53 years. Out of 401 mothers, 204 (50.87%) had other children. All of them were married and lived with their spouses. The details of the socio-demographic characteristics of the participants have been shown in Table 2.

**Table 2:** Demographic characteristics of participants (*n* = 401)

Variable		N (%)	Mean ± standard deviation (Minimum–maximum)
Age (years)	-	-	31.77 ± 6.02 (16–53)
Education level	Postgraduate academic level	135	-
	Undergraduate academic level	80	-
	High-school diploma	110	-
	Less than high-school diploma	76	-
Past history	Yes	(58.20%) 204	-
	No	(41.79%) 197	-
Type of delivery	Cesarean Section	401 (%100)	-
	Normal Vaginal Delivery	0	-
Infant Sex	Female	(44.88%) 180	-
	Male	(55.11%) 221	-
Gestational age	28 ≥	(5.50%) 51	-
	29–32	(49.37%) 198	-
	33–34	(37.90%) 152	-
Birth weight (gr)	-	-	1550.29 ± 437.99 (670–3110)
Discharge weight (gr)	-	-	1905.77 ± 398.85 (1440–3460)

In the construct validity phase, based on the results, the sample’s KMO and Bartlett's values were sufficient and appropriate, 0.897 and 1758.593, respectively (*P* ≤ 0.001). In this step, 17 items were removed as their shared values ​​were less than 0.2 and their factor loadings were less than 0.3. After Promax rotation, 3 factors (totally 21 items) were extracted: cognitive ability (9 items), technical ability (7 items), and psychological ability (5 items). These factors respectively explained 30.59, 9.62, and 7.28% of the total variance (47.44%) of the concept of the caring ability of mothers with preterm infants. The details of the factor analysis result have been presented in Table 3.

**Table 3:** The result of EFA on the five factors of CAMPIS (*N* = 201)

Factors	Qn. Item	Factor loading	Communalities	M (SD)	Skew (kurtosis)	Eigenvalue	%Variance
Cognitive Ability	12. I feel I take good care of my baby	0.814	0.559	4.47 (0.73)	-1.31 (1.47)	6.92	30.59
	35. I act fast enough when caring for my baby	0.798	0.627	4.10 (0.91)	-0.73 (-0.01)		
	1. I have enough physical ability to take care of my baby	0.776	0.543	4.41 (0.73)	-1.2 (2.06)		
	2. I feel comfortable taking care of my baby at home	0.723	0.445	4.55 (0.70)	-1.43 (1.31)		
	32. Even though my baby is thin and weak, I can hug her properly	0.687	0.464	4.52 (0.73)	-1.31 (0.61)		
	36. Although my baby is preterm and needs special care, I can take care of him alone	0.676	0.59	3.77 (1.18)	-0.69 (-0.48)		
	6. I can balance life plans and care for my baby	0.627	0.367	4.09 (0.85)	-0.76 (-0.08)		
	31. When my baby is crying and restless, I can calm him down	0.567	0.552	4.24 (0.86)	-0.92 (0.70)		
	14. I’m sure the things I do for my child are right	0.492	0.405	4.03 (0.80)	-0.29 (-0.83)		
Knowledge and Skills Abilities	23. If I see signs of a feeding difficulties (not breastfeeding well, lethargy, restlessness and heartburn, vomiting, etc.) I know what to do	0.765	0.516	3.58 (1.09)	-0.47 (-0.40)	2.57	9.62
	8. Given that my baby was born preterm, I am aware of the differences in growth and development (such as neck, rolling, walking, etc.) with other babies	0.745	0.486	3.66 (1.22)	-0.48 (-0.78)		
	7. I have enough information about my baby being preterm and its complications	0.717	0.472	3.56 (1.18)	-0.45 (-0.73)		
	24. I can recognize the signs and symptoms of shortness of breath and cyanosis (lack of oxygen)	0.691	0.46	3.58 (1.14)	-0.53 (-0.37)		
	9. If I have a problem, I know who to ask or where to go	0.685	0.424	3.77 (1.19)	-0.77 (-0.21)		
	10. I can clearly identify my baby's needs	0.588	0.539	3.91 (0.91)	-1.2 (0.70)		
	25. I can feed my baby properly. (Breastfeeding time, volume and method, etc.)	0.536	0.471	4.18 (0.88)	-0.59 (0.15)		
Psychological Ability	18. I'm afraid my baby might be harmed	0.674	0.446	2.45 (1.22)	0.53 (-0.61)	2.05	7.28
	13. Given that my baby is preterm and weak, I am worried about losing him	0.636	0.417	3.23 (1.48)	-0.19 (-1.34)		
	21. I'm under a lot of pressure about taking care of my baby	0.63	0.474	2.81 (1.27)	0.21 (-0.90)		
	17. I do not sleep well because I am worried about my baby's health	0.592	0.306	2.68 (1.28)	0.23 (-0.98)		
	16. Because my baby was born preterm, I worry about the blame and reproach	0.576	0.389	3.70 (1.38)	-0.67 (-0.88)		

*EFA* Exploratory factor analysis, *CAMPIS* Caring ability of Mother with Preterm Infant Scale, *M* Mean, *SD* Standard deviation

Based on CFA indices, this sample has an acceptable fit to the 3 factors model and all of these indices in our study are excellent. The results of model fit indices have been given in Table 4.

**Table 4:** Factors adjustment indexes obtained in CFA of the CAMPIS (*n* = 200)

X2	df	*P*value	X2/df	CFI	IFI	RFI	PNFI	TLI	GFI	RMSEA
360.856	183	< 0.001	1.972	0.933	0.933	0.854	0.873	0.923	0.886	0.059

*CFA* Confirmability factor analysis, *CAMPIS* Caring ability of Mother with Preterm Infant Scale, *CFI* Comparative fit index, *IFI* Incremental fit index, *RFI* Frequency interference Index, *PNFI* Parsimonious Normed Fit Index, *TLI* Tucker-Lewis Index, *GFI* Goodness of Fit Index, *RMSEA* Root Mean Square Error of Approximation

Prior to modeling modification, the goodness of fit measures for the CFA-generated 3-factor model indicated that the model fit but not optimally. To improve the factor structure model, we identified the following item content redundancies: Item 24 (If I see signs of an feeding difficulties, I know what to do) is related to Item 23 (I can recognize the signs and symptoms of shortness of breath and cyanosis), and item 8 (Given that my baby was born preterm, I am aware of the differences in growth and development with other babies.) is related to item 7 (I have enough information about my baby being preterm and its complications.).

Also, item 18 (I'm afraid my baby might be harmed.) is related to item 17 (I do not sleep well because I am worried about my baby's health.) Given the similarities of conceptual meaning, these correlated error terms indicated that these variables may share specific variances.

**Fig. 1:**
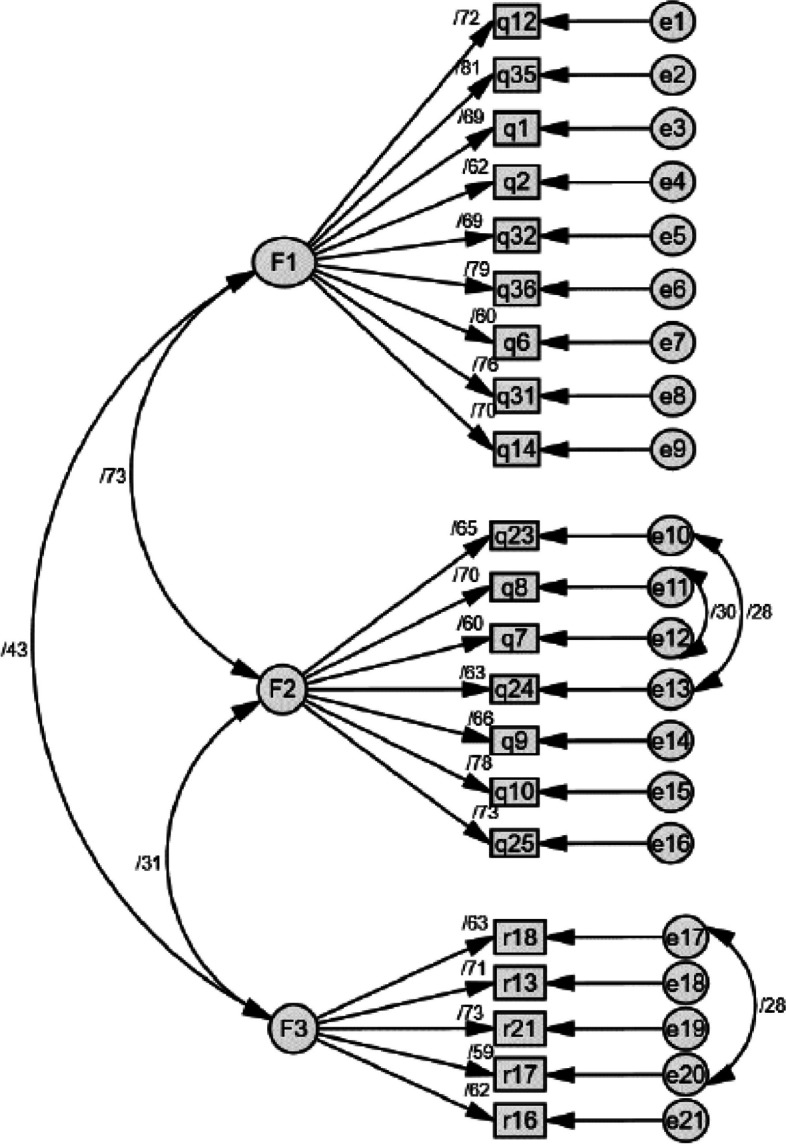
The results of CFA showed that a three-factor model of the care ability of the mothers with preterm infants indicated that the model fitted well. F1: Cognitive Ability; F2: Knowledge and Skills Abilities; F3: Psychological Ability

As shown in Fig. 1, the aforementioned changes improved the goodness of fit of the model. This indicated the model of the caring ability of mothers with preterm infants fits the data (Fig. 1).

The retest method with a time interval of 72 h (the day before discharge and 72 h after discharge) was used in 48 mothers with preterm infants. An ICC value > 0.8 is considered as the acceptable value for stability. In addition, the absolute reliability was evaluated using the standard error of measurement with the formula Standard Error of Measurement (SEM) = SD √ (1- ICC). The details of the responsiveness are reported in Fig. 1.

The results of McDonald Omega coefficient (ω = 0.90), and AIC (0.27) for the 3 factors were excellent. Based on the result, the ICC was 0.91 in the confidence interval of 0.84%-0.95%, which shows that the tool has acceptable measurement stability over time. Based on the SEM results, the absolute reliability was 3.18. This value shows that the scale scores of an individual vary ± 3.18 in repeated tests (Table 5). Based on the results, MDC = 8.78, MDC% = 11.79 and MIC = 0.50, this scale is responsive and interpretable.

**Table 5:** The results of internal consistency and stability of the CAMPIS

	OMEGA	AIC	ICC (CI 0.95%)	SEM
Cognitive Ability	0.89	0.48	0.93 (88%-96%)	1.3
Knowledge and Skills Abilities	0.87	0.46	0.80 (64%-89%)	1.62
Psychological Ability	0.81	0.38	0.88 (93%-79%)	1.36
CAMPIS	0.9	0.27	0.91 (95%-84%)	3.18

*CAMPIS* Caring ability of Mother with Preterm Infant Scale, *AIC* Average Inter-Item Correlation, *ICC* Intraclass correlation coefficients, *CI* Confidence interval, *SEM* Standard error of measurement

## Discussion

The study’s results demonstrated that the concept of caring ability in mothers with preterm infants has three dimensions: cognitive ability, knowledge and skills abilities, and psychological ability. Therefore, CAMPIS is a valid and reliable scale to assess this concept in mothers with preterm infants. This scale includes 21 items, and three factors, cognitive, knowledge and skills, and psychological abilities, which explained 47.44% of the total variance of this concept. The CAMPIS model obtained through EFA was confirmed using CFA.

CAMPIS has three factors: "cognitive ability ", "knowledge and skills abilities" and "psychological ability". The first factor extracted from the scale was "cognitive ability". This factor includes 9 items regarding perception skills, cognitive skills, decision-making skills, movement skills, and attitude, which were extracted with the highest variance (30.59%). Cognitive abilities are the skills that a person needs to do anything from the simplest to the most complex, including perception skills, decision-making skills, movement skills, language skills, and social skills [26]. Cognitive abilities are the link between behavior and the brain structure, which include a wide range of abilities including planning, paying attention, problem solving, performing tasks simultaneously, and cognitive flexibility [27]. In this scale, cognitive ability was defined as the ability of a mother with a preterm baby to use skills which help her process information, think, reason, and solve problems faster and more efficiently. By developing cognitive skills, a person can go through the process of reasoning, decision making, and taking action. In this way, she can make sure that she can perceive the new situation, and perform her role effectively. The findings of a study conducted in 2004 showed that the mother’s possessing the desired attitudinal-cognitive ability had a significant impact on the infant’s health [28].

The second factor extracted from the scale was "technical ability". This factor includes 7 items regarding care knowledge and skills, as well as the ability to apply them; it was extracted with an acceptable variance (9.62%). In the science of care, knowledge is defined as the caregiver's awareness of the care recipient’s needs, strengths, and weaknesses as a unique member [10]. Moreover, in a study conducted by Galvin et al. in 2017, which was conducted with the aim of investigating the analytical features of the concept of readiness for discharge from the hospital, having sufficient knowledge and skills was mentioned as one of the characteristics of readiness for discharge [29]. In this scale, knowledge-skill ability is defined as that the mother’s awareness of the tasks that she must know how to perform in order to provide effective, efficient, and reliable care for her infant. The mothers of preterm infants often have poor knowledge regarding infant care after discharge [30], while the main factor which shows the mother's ability to provide care for the infant is having sufficient knowledge and skills. The mother should know what, how, why, and when to provide care for her infant [30].

The lived experience of the parents with preterm infants showed that in most cases, they are not prepared for the infant’s birth. The birth of a preterm infant puts them in a special situation which requires new care skills [31]. The parents need to acquire these skills that are a prelude to the discharge and transfer of the infant to home [32]. Providing quality care for complex disorders requires specialized caregiving knowledge and skills. When there is a deficiency in these areas, negative psychological impact on the parents and their relationship with their infant can ensue [33]. Studies have shown that having sufficient preparation for discharge and transitional care can help the families of preterm infants with a successful transition from the hospital to the family, reducing the rehospitalization rates [34]. To this end, health care professionals should provide the parents with useful information regarding illness management, strengthen their relationships with the hospital staff, encourage sharing experiences and emotions, and perform home visits [35]. The empowerment method should be appropriate to the caregiver's conditions. It should predict the caregiver's psychological condition as well as his/her emotional and behavioral responses. It should be used to train and support the caregiver in decision-making and managing the caregiving situation, considering his/her experiences, social status, cultural level, and beliefs [36].

The third factor extracted from the scale was "psychological ability". This factor includes 5 items regarding the psychological characteristics of the mothers taking care of preterm infants, with an acceptable variance (7.28%). An individual who is aware of his/her abilities and need for self-dependence, to manage care after discharge, has good psychological ability [29]. It is necessary to achieve psychological ability for an individual to deal with post-discharge challenges and have control over the situation [37]. In the present study, the mother's psychological ability is defined as her possessing the psychological characteristics and mental makeup to feel ready to continue care provision after discharge from the NICU, and to be able to fulfill her caring role successfully. During a traumatic event such as an infant's hospitalization in the NICU, the mothers try to improve their psychological ability [38].

The most mothers of preterm infants are not psychologically prepared for delivery and motherhood [39]. Preterm birth is considered a sudden and unpredictable event, which is accompanied by a feeling of shock and helplessness. The mothers of preterm infants often describe these conditions using terms such as falling to the bottom of a deep well, and being stuck in a whirlwind and storm happening around them; they admit they do not have enough control over what is happening, and lack the ability to take care of their infants [40]. Hospitalization in the NICU and mother-infant separation cause a feeling of inadequacy in the mother [41], which can subsequently affect her psychological ability. This maybe cause using different methods to deal with it. Using ineffective coping methods regarding changes in lifestyle and playing one’s role impacts on mother's caretaking tasks. If the use of ineffective coping methods is not recognized at the right time, and if appropriate measures are not taken, then the nursing diagnosis of coping disability disorder will be imminent [42]. Improved mental health and sufficient psychological ability are an important prerequisite for behavior change, which acts as a link between awareness and action. It can have a moderating role in empowering individuals, leading to positive thoughts, greater self-esteem and goals, more positive emotions and desirable behaviors [43]. Therefore, it is very important to measure the psychological ability of the mother in order for her to acquire the ability to provide quality care for the preterm infant.

Being aware of the caring ability of their mothers, as the main care givers, and designing an intervention to improve their caring ability can prevent negative side effects and help to improve the quality of care. The present study was conducted with the aim of designing and psychometric evaluation of the tool for assessing caring ability in mothers with preterm infants. Since one of the main goals of psychometric evaluation and factor analysis is to maximizing the explained variance by the model, in this research, the variance was 47.44%. Among the scales designed to measure caring ability, regardless of factor analysis extraction method, only one scale, the caring ability scale of the caregivers of cancer patients (67.7%), explains variance more than CAMPIS does [11].

In addition, this CAMPIS had very good internal consistency based on the results of Cronbach's alpha, AIC, and McDonald omega. It should be noted that one of the advantages of this scale is having strong stability based on the ICC value. Another advantage of this study was the assessment of measurement error, responsiveness, and interpretation of the CAMPIS. The results showed that the CAMPIS has the minimum amount of SEM, responsiveness, and interpretability. SEM shows the accuracy of the measurement for each individual, and it is important that this value be small. Responsiveness refers to the ability of a scale to reflect changes in an individual's position over a period. Finally, interpretability refers to the scale's ability to show the significance of changes. These characteristics are an important and necessary part of consensus-based standards for the selection of health measurement tools, which have not been reported in the previous studies on the psychometric characteristics of caring ability.

CAMPIS measures the caring ability of the mother of a preterm infant in the three factors namely ‘Cognitive Ability’ (items: 1–9), ‘knowledge and skills abilities' (items: 10–16), and ‘Psychological Ability’ (items: 17–21). The answer to the items is based on a five-point Likert scale (always (5), most of the time (4), sometimes (3), rarely (2), never (1)). Scoring of items 17,18,19,20 and 21 is reverse so (always (1), most of the time (2), sometimes (3), rarely (4), never (5)). To have an overall score: Sum cognitive ability + sum knowledge and skills abilities + sum psychological ability = Total score. The best way is to calculate the average score for every scale, and compare the results with the average score. In our studies we set the average score for cognitive ability from 9–45, knowledge and skills abilities 7–35, psychological ability 5–25, and overall score 21–105 (Additional file 1: Appendix A). CAMPIS, is a useful scale for professional caregivers and researchers, thanks to its brief items, good variance, reliability, as well as exclusively belonging to this group.

## Conclusion

The findings of this study demonstrated that CAMPIS is a reliable and valid scale with 21 items, which includes the 3 dimensions of attitudinal-cognitive ability, knowledge-skill ability, and psychological ability for measuring the concept of tenacity in family caregivers. Although the exclusiveness of CAMPIS to evaluate the caring ability of mothers with preterm infants is one of the points of strengths in this study, but considering that the samples were selected from the population of Iranian mothers with preterm infants with a gestational age of 34 weeks or less. It should be used with caution in mothers with preterm infants with a gestational age over 34 weeks. Therefore, one of the important limitations was the concern about the generalizability of the findings.

## Limitations and strength

In the construct validity step, data collection was done online. Although the use of online questionnaires, especially during the period of social restrictions due to COVID-19 pandemic, has many advantages, such as the possibility of eliminating the missing data, speeding up the data collection process, and the possibility of collecting data from other provinces and counties. There are also some limitations like self-selection bias, and the lack of interaction with the participants. Furthermore, some qualified mothers were excluded from the study due to illiteracy, the lack of internet access, and the inability to use the phone/laptop to access social networks.

During the item generation step, we considered the different directions of items, but during data reduction, opposite directions were removed. Finally, all items of each factor of CAMPIS have oriented in the same direction, and it is imaginable to create a possibility for response tendency.

One of the problems of self-report scales is that they are subject to the respondent's interpretation of the items, which may not be what the scale designer has intended. In order to reduce this potential problem, continuous testing and modification of the scale has been done. Since ability is a personal matter, the participants were asked not to reveal their names, cities, and the name of the hospitals where their infants were hospitalized. This study has points of strength. One of them is the evaluation of SEM, ICC, responsiveness, and interpretability as important and required items of the COSMIN checklist, which had not been reported previously regarding caring ability scales, but were evaluated in this study.

### Supplementary Information

**Additional file 1: Appendix A.** The last version of "Caring Ability of Mother with Preterm Infant Scale" (CAMPIS).

## Data Availability

The original contributions presented in the study are included in the article. Request access to other supplementary material can be directed to the first or corresponding author.

## Abbreviations

CAMPIS: Caring Ability of Mother with Preterm Infant Scale
EFA: Exploratory factor analysis
CFA: Confirmatory factor analysis
NICU: Neonatal Intensive Care Unit
WHO: World Health Organization
CVR: Content validity ratio
CVI: Content validity index
I-CVI: Item-level content validity index,
S-CVI/Ave: Scale-level content validity index average
KMO: Kaiser–Meyer–Olkin
AIC: Average Inter-Item Correlation
ICC: Intraclass correlation coefficients
MDC: Minimum Detectable Change Analysis
MIC: Minimal Important Change
CFI: Comparative Fit Index
IFI: Incremental Fit Index
RFI: Frequency Interference Index
PNFI: Parsimonious Normed Fit Index
TLI: Tucker-Lewis Index
GFI: Goodness of Fit Index
RMSEA: Root Mean Square Error of Approximation
CI: Confidence Interval
SEM: Standard Error of Measurement

## References

[1] Cao Y, Jiang S, Sun J, Hei M, Wang L, Zhang H (2021). Assessment of neonatal intensive care unit practices, morbidity, and mortality among very preterm infants in China. JAMA Network Open.

[2] Chawanpaiboon S, Vogel JP, Moller A-B, Lumbiganon P, Petzold M, Hogan D (2019). Global, regional, and national estimates of levels of preterm birth in 2014: a systematic review and modelling analysis. Lancet Glob Health.

[3] Lasiuk GC, Comeau T, Newburn-Cook C (2013). Unexpected: an interpretive description of parental traumas’ associated with preterm birth. BMC Pregnancy Childbirth.

[4] Howson C, Merialdi M, Lawn J, Requejo J, Say L. March of dimes white paper on preterm birth: the global and regional toll. March of dimes foundation. 2009:13.

[5] Gerstein ED, Njoroge WF, Paul RA, Smyser CD, Rogers CE (2019). Maternal depression and stress in the neonatal intensive care unit: associations with mother− child interactions at age 5 years. J Am Acad Child Adolesc Psychiatry.

[6] Fowler N, Vo PT, Sisk CL, Klump KL. Stress as a potential moderator of ovarian hormone influences on binge eating in women. F1000Research. 2019;8. 10.12688/f1000research.16895.1 PMC6396839 30854192

[7] Stern DN, Bruschweiler-Stern N. The birth of a mother: How the motherhood experience changes you forever: Basic Books; 1998.

[8] Slade A, Cohen LJ, Sadler LS, Miller M (2009). The psychology and psychopathology of pregnancy. Handbook of Infant Mental Health.

[9] Spinelli M, Frigerio A, Montali L, Fasolo M, Spada MS, Mangili G (2016). ‘I still have difficulties feeling like a mother’: the transition to motherhood of preterm infants mothers. Psychol Health.

[10] Nkongho NO (2003). The caring ability inventory. Measure Nurs Outcomes.

[11] Nemati S, Rassouli M, Ilkhani M, Baghestani AR, Nemati M (2020). Development and validation of ‘caring ability of family caregivers of patients with cancer scale (CAFCPCS)’. Scand J Caring Sci.

[12] Koren PE, DeChillo N, Friesen BJ (1992). Measuring empowerment in families whose children have emotional disabilities: a brief questionnaire. Rehabil Psychol.

[13] Samra HA, McGrath JM, Fischer S, Schumacher B, Dutcher J, Hansen J (2015). The NICU parent risk evaluation and engagement model and instrument (PREEMI) for neonates in intensive care units. J Obstet Gynecol Neonatal Nurs.

[14] Smith V, Young S, Pursley D, McCormick M, Zupancic J (2009). Are families prepared for discharge from the NICU?. J Perinatol.

[15] Barnes CR, Adamson-Macedo EN (2007). Perceived maternal parenting self-efficacy (PMP S-E) tool: development and validation with mothers of hospitalized preterm neonates. J Adv Nurs.

[16] Prasopkittikun T, Tilokskulchai F, Sinsuksai N, Sitthimongkol Y (2006). Self-efficacy in infant care scale: development and psychometric testing. Nurs Health Sci.

[17] Tajalli S, Ebadi A, Parvizy S, Kenner C (2022). Maternal caring ability with the preterm infant: a Rogerian concept analysis. Nurs Forum.

[18] Lindgren B-M, Lundman B, Graneheim UH (2020). Abstraction and interpretation during the qualitative content analysis process. Int J Nurs Stud.

[19] Sharif Nia H, Zareiyan A, Ebadi A (2022). Test Development Process in Health Sciences; Designing and Psychometric properties.

[20] Lawshe CH (1975). A quantitative approach to content validity. Pers Psychol.

[21] Thompson B, Daniel LG (1996). Factor analytic evidence for the construct validity of scores: A historical overview and some guidelines.

[22] Pahlevan Sharif S, Sharif NH (2020). Factor analysis and structural equation modeling with SPSS and AMOS.

[23] Fabrigar L, Wegener Dt, MacCullum R. Strahan, e. J. Evaluating the use of factor analysis in psychological research. Psychological Methods. 1999;4:272-99.

[24] Mulaik SA, James LR, Van Alstine J, Bennett N, Lind S, Stilwell CD (1989). Evaluation of goodness-of-fit indices for structural equation models. Psychol Bull.

[25] Bentler PM (1990). Comparative fit indexes in structural models. Psychol Bull.

[26] Benjamin DJ, Brown SA, Shapiro JM (2013). Who is ‘behavioral’? Cognitive ability and anomalous preferences. J Eur Econ Assoc.

[27] Ones DS, Dilchert S, Viswesvaran C, Salgado JF. Cognitive abilities: Taylor & Francis Group; 2010. 255–75 p.

[28] Rubalcava LN, Teruel GM (2004). The role of maternal cognitive ability on child health. Econ Hum Biol.

[29] Galvin EC, Wills T, Coffey A (2017). Readiness for hospital discharge: a concept analysis. J Adv Nurs.

[30] Hochreiter D, Kuruvilla D, Grossman M, Silberg J, Rodriguez A, Lary L (2022). Improving guidance and maternal knowledge retention after well-newborn unit discharge. Hosp Pediatr.

[31] Amorim M, Alves E, Kelly-Irving M, Silva S (2019). Needs of parents of very preterm infants in Neonatal Intensive Care Units: a mixed methods study. Intensive Crit Care Nurs.

[32] Jing L, Bethancourt C-N, McDonagh T (2017). Assessing infant and maternal readiness for newborn discharge. Curr Opin Pediatr.

[33] Hellesø R, Eines J, Fagermoen MS (2012). The significance of informal caregivers in information management from the perspective of heart failure patients. J Clin Nurs.

[34] Boykova M, Kenner C (2012). Transition from hospital to home for parents of preterm infants. J Perinat Neonatal Nurs.

[35] Lundqvist P, Weis J, Sivberg B (2019). Parents’ journey caring for a preterm infant until discharge from hospital-based neonatal home care—A challenging process to cope with. J Clin Nurs.

[36] Hamilton DL (1968). Personality attributes associated with extreme response style. Psychol Bull.

[37] Carroll Á, Dowling M (2007). Discharge planning: communication, education and patient participation. Br J Nurs.

[38] Aftyka A, Rozalska-Walaszek I, Rosa W, Rybojad B, Karakuła-Juchnowicz H (2017). Post-traumatic growth in parents after infants’ neonatal intensive care unit hospitalisation. J Clin Nurs.

[39] Rossman B, Greene MM, Meier PP. The role of peer support in the development of maternal identity for “NICU moms”. J Obstet Gynecol Neonatal Nurs. 2015;44(1):3–16. 10.1111/1552-6909.12527 PMC4315745 25580732

[40] O'Donovan A, Nixon E (2019). “Weathering the storm:” Mothers’ and fathers’ experiences of parenting a preterm infant. Infant Ment Health J.

[41] Kestler-Peleg M, Lavenda O, Stenger V, Bendett H, Alhalel-Lederman S, Maayan-Metzger A (2020). Maternal self-efficacy mediates the association between spousal support and stress among mothers of NICU hospitalized preterm babies. Early Human Dev.

[42] Ackley BJ, Ladwig GB, Makic MBF, Martinez-Kratz M, Zanotti M. Nursing Diagnosis Handbook, Revised Reprint with 2021–2023 NANDA-I® Updates-E-Book: Elsevier Health Sciences; 2021.

[43] Karademas EC (2006). Self-efficacy, social support and well-being: The mediating role of optimism. Personality Individ Differ.

