# Supplementary material for: Development and psychometric evaluation of "Caring Ability of Mother with Preterm Infant Scale" (CAMPIS): a sequential exploratory mixed-method study
Source: BMC Nurs. 2024 Apr 29;23:297. doi: 10.1186/s12912-024-01960-7 (PMC11057165; doi:10.1186/s12912-024-01960-7)
Supplement: Supplementary file 1 — Additional file 1: Appendix A. The last version of "Caring Ability of Mother with Preterm Infant Scale" (CAMPIS). [file 12912_2024_1960_MOESM1_ESM.docx]

Appendix A

The last version of "Caring Ability of Mother with Preterm Infant Scale" (CAMPIS)

|  | Items | Always | Most of the Time | Sometimes | Rarely | Never |
| --- | --- | --- | --- | --- | --- | --- |
| 1 | I feel I take good care of my baby. |  |  |  |  |  |
| 2 | I act fast enough when caring for my baby. |  |  |  |  |  |
| 3 | I have enough physical ability to take care of my baby. |  |  |  |  |  |
| 4 | I feel comfortable taking care of my baby at home. |  |  |  |  |  |
| 5 | Even though my baby is thin and weak, I can hug her properly. |  |  |  |  |  |
| 6 | Although my baby is preterm and needs special care, I can take care of him alone. |  |  |  |  |  |
| 7 | I can balance life plans and care for my baby. |  |  |  |  |  |
| 8 | When my baby is crying and restless, I can calm him down. |  |  |  |  |  |
| 9 | I am sure the things I do for my child are right. |  |  |  |  |  |
| 10 | If I see signs of an eating disorder (not breastfeeding well, lethargy, restlessness and heartburn, vomiting, etc.) I know what to do. |  |  |  |  |  |
| 11 | Given that my baby was born preterm, I am aware of the differences in growth and development (such as neck, rolling, walking, etc.) with other babies. |  |  |  |  |  |
| 12 | I have enough information about my baby being preterm and its complications. |  |  |  |  |  |
| 13 | I can recognize the signs and symptoms of shortness of breath and bruising (lack of oxygen). |  |  |  |  |  |
| 14 | If I have a problem, I know who to ask or where to go. |  |  |  |  |  |
| 15 | I can clearly identify my baby’s needs. |  |  |  |  |  |
| 16 | I can feed my baby properly. (Breastfeeding time, volume and method, etc.) |  |  |  |  |  |
| 17 | I am afraid my baby might be harmed. |  |  |  |  |  |
| 18 | Given that my baby is preterm and weak, I am worried about losing him. |  |  |  |  |  |
| 19 | I am under a lot of pressure about taking care of my baby. |  |  |  |  |  |
| 20 | I do not sleep well because I am worried about my baby's healthy. |  |  |  |  |  |
| 21 | Because my baby was born preterm, I worry about the blame and [reproach](https://abadis.ir/entofa/reproach/). |  |  |  |  |  |
